# The ratio of the maximum density values: a new method for predicting hemorrhagic transformation in acute ischemic stroke patients undergoing mechanical thrombectomy

**DOI:** 10.3389/fneur.2024.1357689

**Published:** 2024-03-22

**Authors:** Xiaohong Qiao, Fuhao Zheng, Manman Wei, Zhenming Zhao

**Affiliations:** ^1^Department of Neurology, Weihai Central Hospital Affiliated to Qingdao University, Weihai, China; ^2^Department of Neurointervention, Weihai Central Hospital Affiliated to Qingdao University, Weihai, China

**Keywords:** acute ischemic stroke, non-enhanced cranial CT, postinterventional cerebral hyperdensities, hemorrhagic transformation, mechanical thrombectomy

## Abstract

**Background:**

It is challenging yet critical to differentiate between hemorrhagic transformation (HT) and contrast extravasation on non-contrast-enhanced computed tomography (NCCT) scans following mechanical thrombectomy (MT) in patients with acute ischemic stroke. We propose a new method called the ratio of maximum density values (RMDV) to minimize the confusion of contrast extravasation and to evaluate the diagnostic significance of RMDV in predicting HT on immediate post-interventional NCCT scans.

**Methods:**

We conducted a retrospective analysis of the prospective patients’ database who received MT for acute ischemic stroke caused by occlusion of the intracranial large artery and showed postinterventional cerebral hyperdensities (PCHDs) on NCCT scans immediately after MT. Based on the subsequent NCCT scans, we divided patients with PCHDs into the HT and the non-HT groups. The clinical characters and radiological details were collected and compared to the two groups. We assessed the ability of RMDV >1 to predict HT by analyzing the receiver operating characteristic curve.

**Results:**

One hundred and three patients showed PCHDs; 58 (56.31%) were classified as HT, while 45 (43.69%) were classified as non-HT. The only notable distinction between the two groups was the proportion of RMDV >1 in the HT group. The correlation between HT and RMDV >1 with an area under the curve of 0.826 (95% confidence interval, 0.739 to 0.894). The sensitivity, specificity, positive, and negative predictive values of RMDV >1 on NCCT for predicting HT were 89.66, 75.56, 82.54, and 85.00%, respectively.

**Conclusion:**

The utilization of RMDV >1 on immediate NCCT scans after MT can predict early HT with good sensitivity and specificity.

## Introduction

Mechanical thrombectomy (MT) has emerged as the optimal therapeutic approach for patients suffering from acute ischemic stroke (AIS) due to large vessel occlusion (LVO). Currently, MT achieves successful recanalization in approximately 80–90% of cases. However, only 50% of patients have a favorable prognosis, while futile recanalization occurs in 30–40% of cases (1, 2). Hemorrhagic transformation (HT) is one of the factors contributing to futile recanalization (3), with an incidence rate of 30–50% and a mortality rate of approximately 3% (4, 5). Both asymptomatic and symptomatic HT have been found to be associated with poorer functional outcomes (5). Therefore, early identification of HT after MT is crucial to predict prognosis and determine the optimal timing for starting antithrombotic therapy (6).

It widely utilizes non-contrast-enhanced computed tomography (NCCT) to evaluate postoperative HT. Postinterventional cerebral hyperdensities (PCHDs), which may contain hemorrhagic products and contrast agents, are commonly observed on NCCT scans after interventions and have been documented with a strong association with worse clinical outcomes (7). However, the differentiation between HT and contrast extravasation in the early stages after MT on postinterventional NCCT scans can be challenging due to the similar CT density values of blood product and iodine contrast (8).

The frequency of hyperdense lesions following MT on NCCT has been reported to range from 32 to 84% (9, 10), significantly higher than the rates of postoperative HT, ranging from 35 to 49%. While about 30% of those who were judged HT using postoperative NCCT alone actually were lots in contrast extravasation (11).

In theory, HT lesions contain blood products and contrast agents. The density of HT is greater than the density of the contrast extravasation on NCCT scans immediately after MT. For the reasons mentioned above, we introduce a new method called the ratio of the maximum density values (RMDV) to minimize the potential confusion of the contrast agent. The purpose of this study was to evaluate the effectiveness of RMDV in predicting HT on immediate post-interventional NCCT scans.

## Methods

### Study population

Between January 2022 and July 2023, we retrospectively analyzed the prospective database of patients with AIS caused by LVO and underwent MT at our comprehensive stroke center. Patients were considered eligible if the following enrollment criteria were fulfilled: (1) age ≥18 years; (2) AIS due to LVO; (3) endovascular recanalization using MT within 24 h after symptom onset; (4) patients underwent NCCT immediately after MT and 24 ± 4 h post-procedural. We excluded patients with incomplete images or clinical data. The Ethics Committee of the Weihai Central Hospital Affiliated to Qingdao University approved our study (Ethics approval number: LL-2024-007).

### Clinical data

We collected each patient’s data, including baseline characteristics, vascular risk factors, use of intravenous thrombolytic, baseline NIHSS score, baseline ASPECTS/pc-ASPECTS, site of intracranial occlusion, procedural time metrics, revascularization status, and functional outcomes. The TOAST classification was used to categorize stroke subtypes. Stroke severity on admission was obtained using the National Institutes of Health Stroke Scale (NIHSS). At 90 days, the prognosis was assessed using the modified Rankin Scale (mRS) score. A score of 0–2 indicated a favorable prognosis, while a score of 3–6 indicated a poor prognosis. MT was carried out by experienced interventionists under local or general anesthesia or conscious sedation. The strategy and devices used for MT were chosen at the discretion of the interventionists. The modified thrombolysis in cerebral infarction (mTICI) score was used to assess the recanalization status after MT. Successful vessel recanalization was defined as achieving a mTICI score of 2b-3.

### Imaging protocol and imaging analysis

A head NCCT scan (GE Discovery CT750HD; GE Healthcare, Chicago, IL) was performed immediately and 24 ± 4 h after MT in all patients. The parameters for NCCT were 100 kV, 120 mAs, and a section thickness of 5 mm. PCHDs were identified as areas of increased density within the brain parenchymal on NCCT scans immediately after MT. The density of these regions was found to be at minimum 5 Hounsfield units (HU) greater than the unaffected contralateral side. The maximum density values of PCHDs and the venous sinuses were manually measured on the NCCT scans immediately after MT. Initially, measurements were taken at multiple points to identify the areas with the highest density among all PCHDs and venous sinuses. A circular or elliptical region of interest (ROI) was then selected, limited to the highest density area, and it was preferred to be as large as possible. The ROI for the sinus area should not include adjacent skulls or artifacts. The ratio of the maximum density values (RMDV) was defined as the maximum density values of PCHDs over the maximum density values of the venous sinuses. We hypothesize that an RMDV >1 would indicate HT, while an RMDV ≤1 would indicate contrast extravasation.

Contrast extravasation was defined as PCHDs eliminated on the 24 h follow-up NCCT; otherwise, they were considered HT.

### Statistical analysis

Descriptive analyses for continuous variables were presented as median with mean ± SD or interquartile range (IQR), while categorical variables were provided as frequencies and percentages. The clinical data were compared using different statistical tests. The chi-square test was used for categorical data, the Mann–Whitney *U* test for non-normally distributed continuous data, and the student’s *t*-test for normally distributed continuous data. Prognostic performance of RMDV >1 in predicting HT was evaluated by creating a receiver operating characteristic (ROC) curve and calculating the area under the curve (AUC). Additionally, the sensitivity, specificity, positive predictive value, and negative predictive value of RMDV >1 for predicting HT were calculated. *p* < 0.05 was considered statistically significant. All data were performed by SPSS 26.0 (IBM Corp., Armonk, NY, United States).

## Result

### Characteristics of total population

One hundred sixty-two patients diagnosed with AIS underwent MT in our center during the research period. Among these patients, 18 patients were not included in the study for the reasons described below: (1) three patients were excluded due to the presence of severe artifacts on the CT scans, (2) eight patients were excluded since they did not have immediate NCCT scans after MT, and (3) seven patients were excluded because they did not have NCCT scans 24 ± 4 h post-procedural. Finally, a total of 144 patients were ultimately enrolled. Among these patients, 94 (65.28%) were male and the remaining 50 (34.72%) were female. The ages of the patients ranged from 28 to 90 years.

According to the following NCCT scans, 60 (41.67%) patients had a subsequent HT, and 8 (5.56%) were symptomatic. 103 (71.53%) showed PCHDs on NCCT scans immediately after MT. Among the HT after MT,58 (96.67%) showed PCHDs immediately after MT, and 2 (3.33%) showed non-PCHDs.

### Comparison of clinical features between the two groups

One hundred and three patients showed PCHDs on NCCT scans immediately after MT. The demographic and clinical information for PCHDs patients is shown in Table 1. We divided patients with PCHDs into two categories based on the subsequent NCCT scans: the HT and non-HT groups. Among the patients, 58 (56.31%) were classified as HT, while 45 (43.69%) were classified as non-HT. No statistically significant disparities were observed between the two groups regarding age, gender, intravenous thrombolytic treatment, prior stroke history, smoking habits, use of anticoagulants, NIHSS scores, procedural time metrics, mTICI scores, and other variables (*p* > 0.05). The HT group had a hypertension prevalence of 58.62% (34/58), compared to 33.33% (15/45) in the non-HT group (*p* = 0.062). Similarly, the prevalence of diabetes in the HT group was 34.48% (20/58), whereas it was 17.78% (8/45) in the non-HT group (*p* = 0.059). Compared to the non-HT group, the HT group had a significantly higher rate of RMDV >1 (*p* < 0.001). Table 1 provides details.

**Table 1:** The clinical characteristics of patients with PCHDs on NCCT.

	PCHDs (*n* = 103)	HT group (*n* = 58)	No-HT group (*n* = 45)	*χ*^2^/*t*/*Z*	*p*-value
Age (mean) (years)	68.90 ± 11.53	70.72 ± 9.19	66.56 ± 13.74	−1.754	0.084
Sex (male, %)	67 (65.05)	34 (58.62)	33 (73.33)	2.413	0.120
Hypertension, *n* (%)	49 (47.57)	34 (58.62)	15 (33.33)	3.484	0.062
Diabetes, *n* (%)	28 (27.18)	20 (34.48)	8 (17.78)	3.572	0.059
Previous stroke, *n* (%)	23 (22.33)	15 (25.86)	8 (17.78)	0.955	0.328
Atrial fibrillation, *n* (%)	54 (52.43)	32 (55.17)	22 (48.89)	0.401	0.527
Smoking, *n* (%)	41 (39.81)	21 (36.21)	20 (44.44)	0.718	0.397
Coronary heart disease	26 (25.24)	18 (31.03)	8 (17.78)	2.360	0.124
Hyperlipidemia	32 (31.07)	17 (29.31)	15 (33.33)	0.191	0.662
Anticoagulant use, *n* (%)	11 (10.68)	5 (8.62)	6 (13.33)	0.199	0.655
Antiplatelet drug, *n* (%)	14 (13.59)	9 (15.52)	5 (11.11)	0.419	0.518
Intravenous thrombolytic, *n* (%)	48 (46.60)	30 (51.72)	18 (40.00)	1.400	0.237
**Occluded arteries, *n* (%)**					
MCA isolated	66 (64.08)	41 (70.69)	25 (55.56)	2.521	0.112
ICA isolated or in tandem with MCA	27 (26.21)	14 (24.14)	13 (28.89)	0.296	0.587
Posterior circulation	11 (10.68)	3 (5.17)	8 (17.78)	3.003	0.083
Baseline systolic blood pressure (mmHg)	150.03 ± 21.18	150.78 ± 20.33	149.07 ± 22.43	−0.404	0.687
Baseline diastolic blood pressure (mmHg)	87.26 ± 14.21	88.97 ± 13.92	85.07 ± 14.43	−1.387	0.168
Baseline NIHSS score, median (IQR)	16 (12–20)	16 (14–20)	16 (10–22)	−0.626	0.531
ASPECTS/pc-ASPECTS, median (IQR)	8 (7, 9)	8 (7, 9)	8 (7, 9)	−1.051	0.293
**TOAST classification, *n* (%)**					
Cardiac embolism	54 (52.43)	34 (58.62)	20 (44.44)	2.042	0.153
Large-artery atherosclerosis	46 (44.66)	23 (39.66)	23 (51.11)	1.346	0.246
Onset to recanalization, median (IQR) (min)	240 (180–300)	240 (180–300)	240 (150–300)	−0.097	0.923
Procedure time (IQR) (min)	110 (83–150)	110 (82–140)	100 (82–175)	−0.063	0.950
Time interval between reperfusion to the first post-CT (IQR) (min)	20 (18, 22)	20 (19, 23)	20 (18, 22)	−1.162	0.245
mTICI ≥2b, *n* (%)	83 (80.58)	44 (75.86)	39 (86.67)	1.891	0.169
>3 stent pass attempts, *n* (%)	34 (33.01)	21 (36.21)	13 (28.89)	0.614	0.433
Intraoperative balloon dilatation, *n* (%)	20 (19.42)	8 (13.79)	12 (26.67)	2.884	0.089
Intraoperative tirofiban use	13 (12.62)	5 (8.62)	8 (17.78)	1.927	0.165
3 months mRS >2, *n* (%)	64 (62.14)	40 (68.97)	24 (53.33)	2.632	0.105
Mortality rate, *n* (%)	18 (17.48)	13 (22.41)	5 (11.11)	2.245	0.134
RMDV >1, *n* (%)	63 (61.17)	52 (89.66)	11 (24.44)	45.364	<0.001

MCA, middle cerebral artery; ICA, internal carotid artery; IQR, interquartile range; NIHSS, National Institutes of Health Stroke Scale; mRS, modified Rankin Score; mTICI, modified thrombolysis in cerebral infarction; ASPECTS, Alberta stroke program early CT score; pc-ASPECTS, posterior circulation Alberta Stroke Program Early CT Score.

### RMDV >1 could predict HT

**Figure 1:**
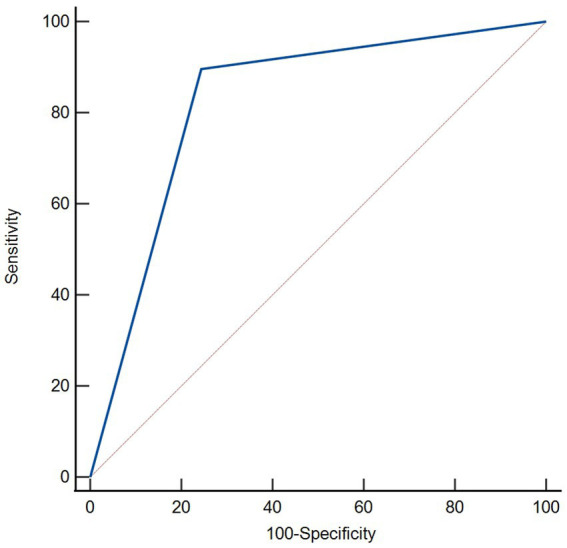
Receiver operating characteristic curves were used for predicting value of RMDV >1 for HT on immediate NCCT after MT. RMDV >1 was a predictor of HT with statistical significance (area under curve of 0.826; 95% confidence interval: 0.739 to 0.894, *p* < 0.001).

**Figure 2:**
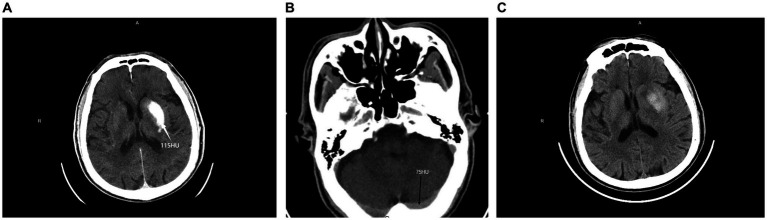
PCHDs on NCCT images obtained immediately after MT **(A,B)** and 24 h follow-up NCCT images **(C)** in one patient. It shows hyperdensity in the left basal ganglia; the maximum density value was 115 HU **(A)**, while the maximum density value of the transverse sinus was 75 HU **(B)**, RMDV>1 HT was confirmed by 24 h follow-up NCCT **(C)**.

The ROC curve analysis revealed a significant correlation between HT and RMDV >1. The area under the curve (AUC) was 0.826 with a 95% confidence interval of 0.739 to 0.894. With a specificity of 75.56% and sensitivity of 89.66%, the Youden index was 0.65. The positive and negative predictive values were 82.54 and 85.00%, respectively. The detailed results were shown in Figure 1. Representative images of PCHDs after MT to predict HT was shown in Figure 2.

## Discussion

To the best of our knowledge, this is the first investigation that provide the evidence that RMDV >1 can effectively differentiate HT from contrast extravasation on the postinterventional NCCT scans immediately after MT.

After MT, HT and contrast extravasation represent different degrees of blood-brain barrier damage caused by ischemia and reperfusion therapy. When this damage is limited to endothelial permeability, it results in contrast extravasation and the absence of blood products (12). Typically, the contrast agent disappears within 24 h (13). Prolonged ischemia or delayed reperfusion can worsen the disruption of the blood-brain barrier, resulting in HT. The similarity between contrast extravasation and HT has caused a lack of consensus among physicians regarding the diagnosis and classification of intracerebral hemorrhage on CT scans (14, 15).

In the present investigation, the prevalence of PCHDs was found to be 71.53%. At the same time, HT was observed in 41.67% of the cases, similar to previous reports. To distinguish between contrast extravasation and HT, it is important to note that the contrast agent can be observed in both the blood vessels and PCHDs. Therefore, the ratio of CT density values between the PCHDs and the blood vessels can be used to mitigate the influence of contrast agents and improve the accuracy of identifying HT. On postoperative NCCT scans, the venous sinuses are more prominently visible compared to the intracranial arteries. The transverse sinuses, in particular, are broader and provide more precise measurements. Therefore, we selected the density of the transverse sinus as the ratio.

The findings of this study indicate that utilizing this ratio can substantially enhance the sensitivity and specificity of diagnosing HT. RMDV can be easily estimated and does not require any additional equipment or software programming. It can help with the clinical care of patients following MT and offer clinicians useful prognostic information. For instance, patients with RMDV >1 should be carefully monitored for blood pressure control and antiplatelet or anticoagulation treatments should be delayed or avoided.

Previously, there were several other methods to identify HT using NCCT. Xu et al. (16) utilized NCCT to establish the metal hyperdensity sign with a density exceeding 90 HU and to identify HT in the basal ganglia region after MT. The sensitivity of the sign was found to be 88.2%, and the specificity was 90.5%. However, it is essential to note that the study only focused on lesions in the basal ganglia region and limited its broader application. In addition, Cai et al. (17) found that the presence of the metal hyperdensity sign was observed in only 28% of all patients with lesions in the brain parenchyma area. Dual-source CT can potentially differentiate brain tissue, hemorrhage, and iodine agents. However, it is worth mentioning that the outcomes of various studies exhibit significant variations. Bonatti et al. (18) found that dual-source CT had a 100% positive predictive value for HT following acute endovascular treatment. However, the sensitivity was only 20%. Cai et al. (17) also found that only 82% of the HT diagnosed by dual-source CT was confirmed by 24 h head CT. The study also indicated that dual-energy CT may not be superior to non-contrast CT in predicting subsequent hemorrhage after MT. Therefore, the potential applications of dual-energy CT still require further validation.

Many previous studies have explored the risk factors for hemorrhagic transformation after MT. Factors such as hypertension, diabetes, >3 stent pass attempts and operation time have been reported to be correlated with hemorrhagic transformation (19–21). No significant statistical differences were found in the baseline data, operation details, and prognosis between the two groups of patients who developed PCHDs after MT in our study. However, the HT group had more patients with diabetes and hypertension than the non-HT group. This difference was not statistically significant, considering the small sample size of our study.

### Limitations

Our study has several limitations. Firstly, the study was conducted retrospectively, utilizing a limited sample size from a single center. Although the data were established prospectively, the retrospective nature of this design could introduce selection bias and restrict the generalizability of our findings. Multicenter, prospective studies are needed to confirm our results. Secondly, we identified HT according to NCCT scans, while many other methods, such as SWI, can predict HT more precisely. However, it is worth mentioning that one patient can have multiple hyper-densities. Our study selected the maximum density of PCHDs without further analyzing the sites or calculating every site by RMDV. Furthermore, the impact of contrast media on the risk of HT has been reported in previous studies. Unfortunately, we could not obtain information on the amount of contrast media used in some of our patients, which restricted our ability to evaluate this factor fully.

## Conclusion

In this study, using RMDV >1 to determine early hemorrhagic transformation is a simple method with good sensitivity and specificity. This method can assist clinicians in making early judgments and taking appropriate measures to prevent catastrophic and severe hemorrhage.

## Data availability statement

The raw data supporting the conclusions of this article will be made available by the authors, without undue reservation.

## Ethics statement

The studies involving humans were approved by the Ethics Committee of the Weihai Central Hospital Affiliated to Qingdao University. The studies were conducted in accordance with the local legislation and institutional requirements. The hospital ethics committee waived the requirement of written informed consent for participation from the participants or the participants’ legal guardians/next of kin because this paper is a retrospective study.

## Author contributions

XQ: Data curation, Methodology, Software, Writing – original draft, Writing – review & editing. FZ: Conceptualization, Methodology, Supervision, Writing – original draft, Writing – review & editing. MW: Data curation, Methodology, Writing – original draft. ZZ: Investigation, Methodology, Writing – original draft.
